# Anesthetic management for cesarean section in two parturient with ascending aortic aneurysm: a case-based discussion

**DOI:** 10.1186/s12871-024-02553-2

**Published:** 2024-05-06

**Authors:** Ana Vuzdar Trajkovski, Krešimir Reiner, Nikolina Džaja, Gloria Mamić, Mirabel Mažar, Jasminka Peršec, Vicko Gluncic, Anita Lukic

**Affiliations:** 1 Department of Anesthesiology, Perioperative Management and Intensive Care in Gynecology and Obstetrics, Clinic of Anesthesiology, Reanimatology, Intensive Care and Pain Therapy, University Clinical Hospital Centre Zagreb, Kišpatićeva 12, Zagreb, 10000 Croatia; 2 Department of Anesthesiology and Intensive Care in Cardiac and Vascular Surgery, Clinic of Anesthesiology, Reanimatology, Intensive Care and Pain Therapy, University Clinical Hospital Centre Zagreb, Kišpatićeva 12, Zagreb, 10000 Croatia; 3 Clinic of Anesthesiology, Reanimatology and Intensive Care Medicine, University Clinical Hospital Dubrava, Avenija Gojka Šuška 6, Zagreb, 10000 Croatia; 4 Department of Anesthesia, Advocate Illinois Masonic Medical Center, 836 W Wellington Ave, Chicago, IL 60657 USA; 5 Department of Anesthesia, Intensive Medicine, and Reanimation, Varazdin General Hospital, 1 I. Mestrovica Street, Varazdin, 42 000 Croatia; 6 University North, Ul. 104. Brigade 3, Varazdin, 42 000 Croatia; 7 Bjelovar University of Applied Sciences, Nursing Studies, 4 Eugena Kvaternika Square, Bjelovar, HR-43000 Croatia

**Keywords:** Anesthesia, Cesarean section, Ascending aortic aneurysm, Aortic dissecting aneurysm, Ruptured aneurysm, Remifentanil

## Abstract

**Background:**

The anesthetic management of parturients with ascending aortic aneurysm for cesarean section can be particularly challenging, primarily because of increased risk for aortic dissection or aneurysm rupture.

**Case presentation:**

We present some aspects of the anesthetic management of two parturients with ascending aortic aneurysm for cesarean sections; amongst, the use of remifentanil with its effects on patient and newborn. We emphasize the importance of a cardio-obstetric team in the context of preoperative planning of such patients. Also, we reviewed some literature on the anesthetic management with its effect on peri-operative hemodynamic stability.

**Conclusion:**

Maintaining hemodynamic stability is paramount in the prevention of the rupture or dissection of ascending aortic aneurysm during labor of parturient.

## Background

The anesthetic management of parturients with aortic dilation poses significant challenges. While there are two primary options – neuraxial and general anesthesia –, several specific characteristics regarding aortic dilatation must be taken into consideration for proper decision-making.

In this report, we present the anesthetic management of two patients with ascending aortic aneurysm for cesarean section under general anaesthesia. Given the rarity of ascending aortic aneurysms during pregnancy and the limited availability of reports and no recommendations regarding anesthesia in such cases, we consider our experience holds valuable insights for obstetric anesthesiologists. Therefore, we present these cases and the review of literature within a case-based discussion framework.

## Case presentation

### Case 1

**Fig. 1:**
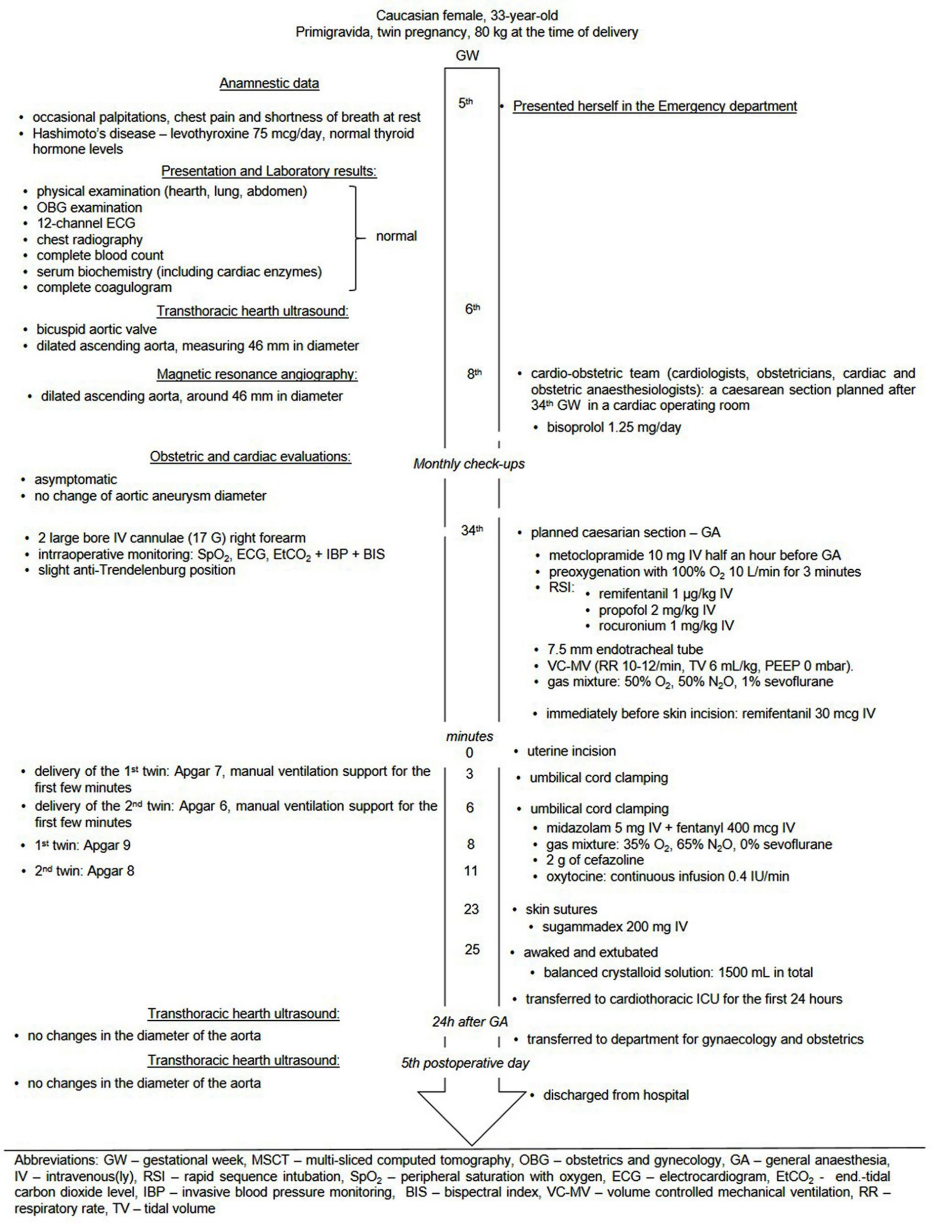
Flowchart of the first parturient with ascending aortic aneurysm

Our first patient, a 33-year-old primigravida with a twin pregnancy, presented herself in the emergency department during the 5th gestational week (GW) due to occasional palpitations, chest pain and shortness of breath during rest (Fig. 1).

**Fig. 2:**
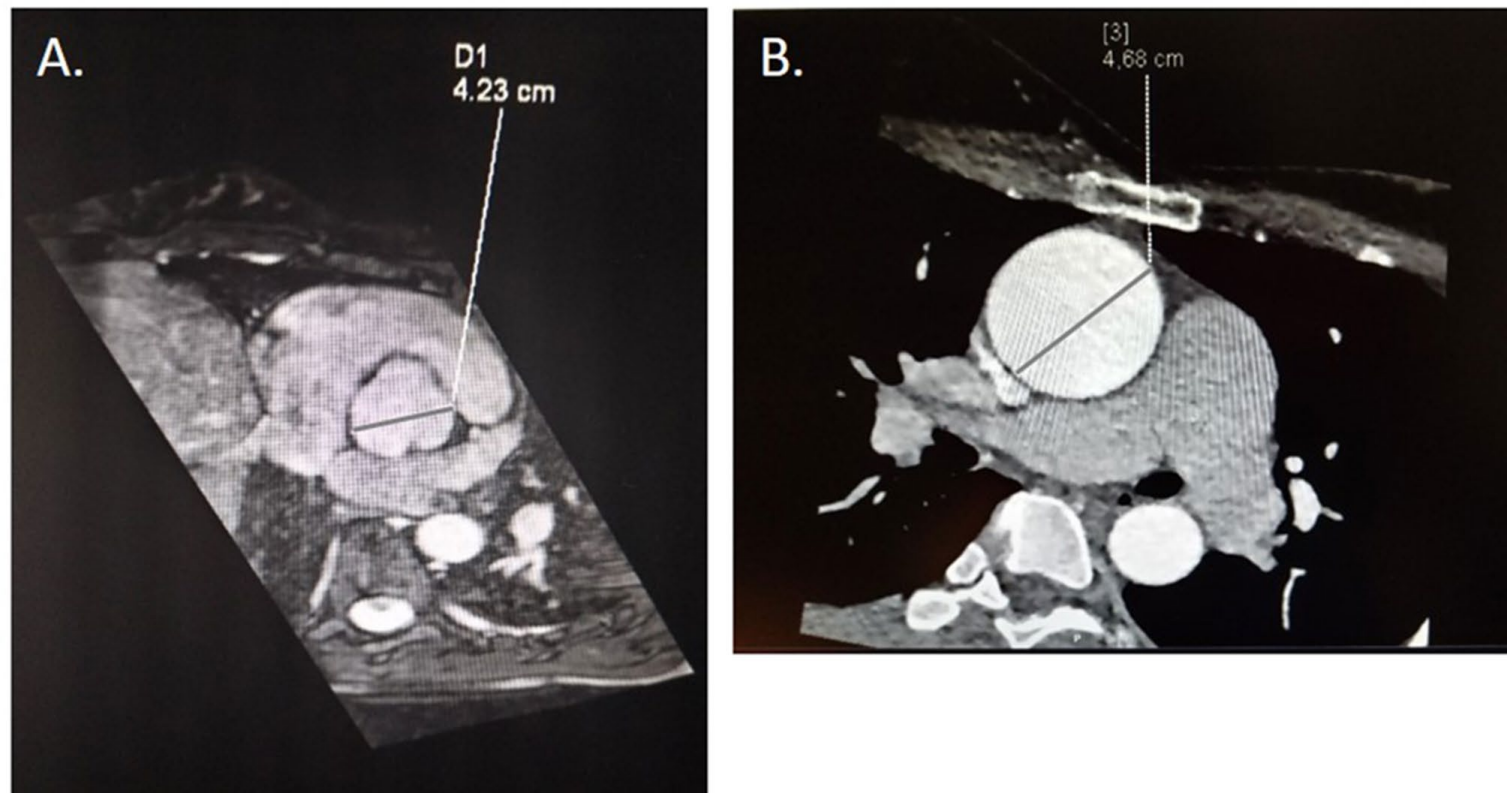
Ascending aortic aneurysms. (**A**): Magnetic resonance aortography of the first parturient. (**B**): Multislice computed tomography aortography of the second parturient

As the physical and obstetric examinations, all laboratory findings, and the chest radiograph and electrocardiogram were normal, the patient was referred for transthoracic ultrasound (US). This assessment took place a few days later and showed a bicuspid aortic valve along with an ascending aorta aneurysm measuring 46 mm in diameter. Subsequently, the diagnosis was confirmed with magnetic resonance angiography of the thoracic aorta conducted during the 8th GW (Fig. 2A).

The patient was prescribed bisoprolol 1.25 mg/day. A multidisciplinary cardio-obstetric team, consisting of cardiologists, obstetricians and cardiac and obstetric anesthesiologists opted for a scheduled cesarean section following the completion of the 34th GW. Throughout her pregnancy, the patient remained asymptomatic, and there were no changes in the diameter of aortic aneurysm observed during monthly obstetric and cardiac evaluations.

**Fig. 3:**
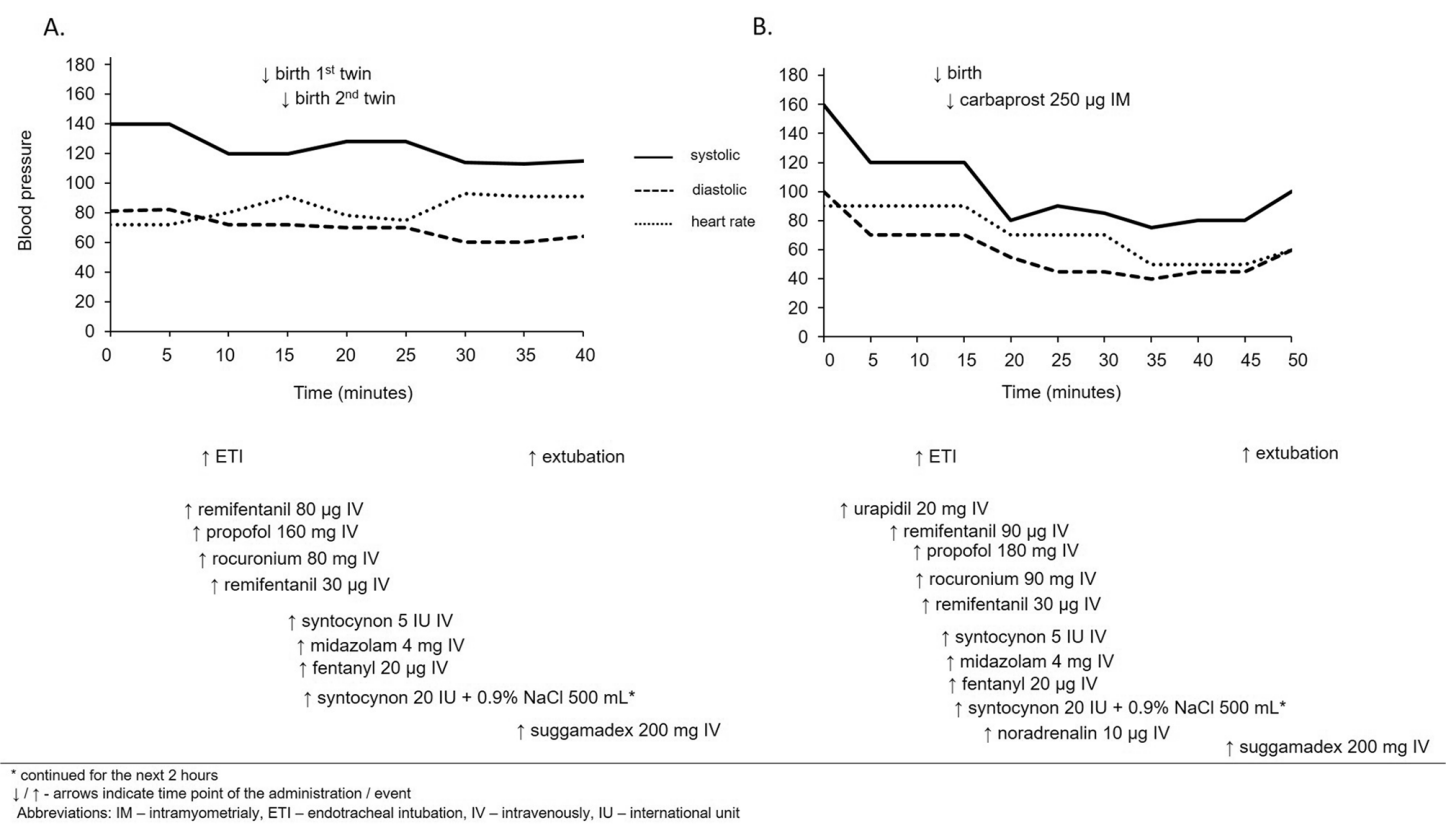
Intraoperative haemodynamics. (**A**): The first parturient. (**B**): The second parturient

The cesarean section was conducted in a cardiac operating room, given concerns that aortic aneurysm might dissect or rupture during the procedure. Although the benefits of regional anesthesia for cesarean section were explained to the patient, it was decided to proceed with general anesthesia due to the patient’s anxiety regarding associated risks. Prior to induction, the patient was prepared by placement of IV lines and intraoperative monitoring for anesthesia (Fig. 1). Standard aspiration prophylaxis was administered, blood products were prepared in accordance with a massive transfusion protocol and ready for immediate use if necessary. The neonatology team was on standby and informed of a likelihood of neonatal respiratory depression and low Apgar scores due to placental transfer of remifentanil. General anesthesia was conducted as shown in Fig. 1, and the patient was warmed with a warming blanket. The first twin was delivered 3 min after uterine incision, followed by the second twin 3 min later. Both twins were female, with the first weighting 1590 g and measuring 44 cm in length, and the second weighting 1780 g and measuring 45 cm in length. Initially, both twins had low 1st -minute-Apgar scores and were supported by manual ventilation for the first minutes, but their Apgar scores were improved in the 5th minute (Fig. 1). After clamping both umbilical cords, anesthesia was deepened with midazolam, and the inspiratory oxygen level was reduced while the amount of nitrous oxide in the fresh gas flow was increased to maintain anesthesia. Sevoflurane was discontinued duet to its potential imapct on uterine tone (Fig. 1). The patient received antibiotic prophylaxis, and a continuous infusion of synthetic oxytocin was initiated (Fig. 1) The intraoperative course, including haemodynamics and bispectral (BIS) index values between 40 and 60, was uneventful (Fig. 3A), with an estimated total blood loss of 1 L.

Following the placement of skin sutures, the neuromuscular block was reversed and the patient recovered from anesthesia. She was extubated in the operating room, and then hemodynamically stable and respiratory sufficient transferred to the cardiothoracic intensive care unit (ICU) for the first 24 h. Transthoracic ultrasound of the aorta performed on the 1st and 5th postoperative days showed no changes in diameter. Therefore, the patient was allowed for discharge from hospital.

### Case 2

The second patient was a 37-year-old woman from a family now known for a heterozygous pathogenic variant of FLNA (filamin A). She was diagnosed with mild mitral- and aortic-valve regurgitations since her youth. Both regurgitations were detected during the diagnostic workup of heart murmur. A mild dilatation of the ascending aorta was discovered by cardiac ultrasound examination following two spontaneous miscarriages during the first trimester. Subsequent multi-slice computed tomography (CT) aortography confirmed the presence of an ascending aortic aneurysm, measuring 46 mm in diameter. Genetic testing was conducted thereafter and a single pathogenic variant in FLNA was identified. Following this discovery, genetic testing extended to all family members, revealing identical mutations in the patient’s mother and younger sister. A little later, the patient’s mother passed away suddenly at the age of 59, and autopsy revealed a dissection of the thoracic aorta.

**Fig. 4:**
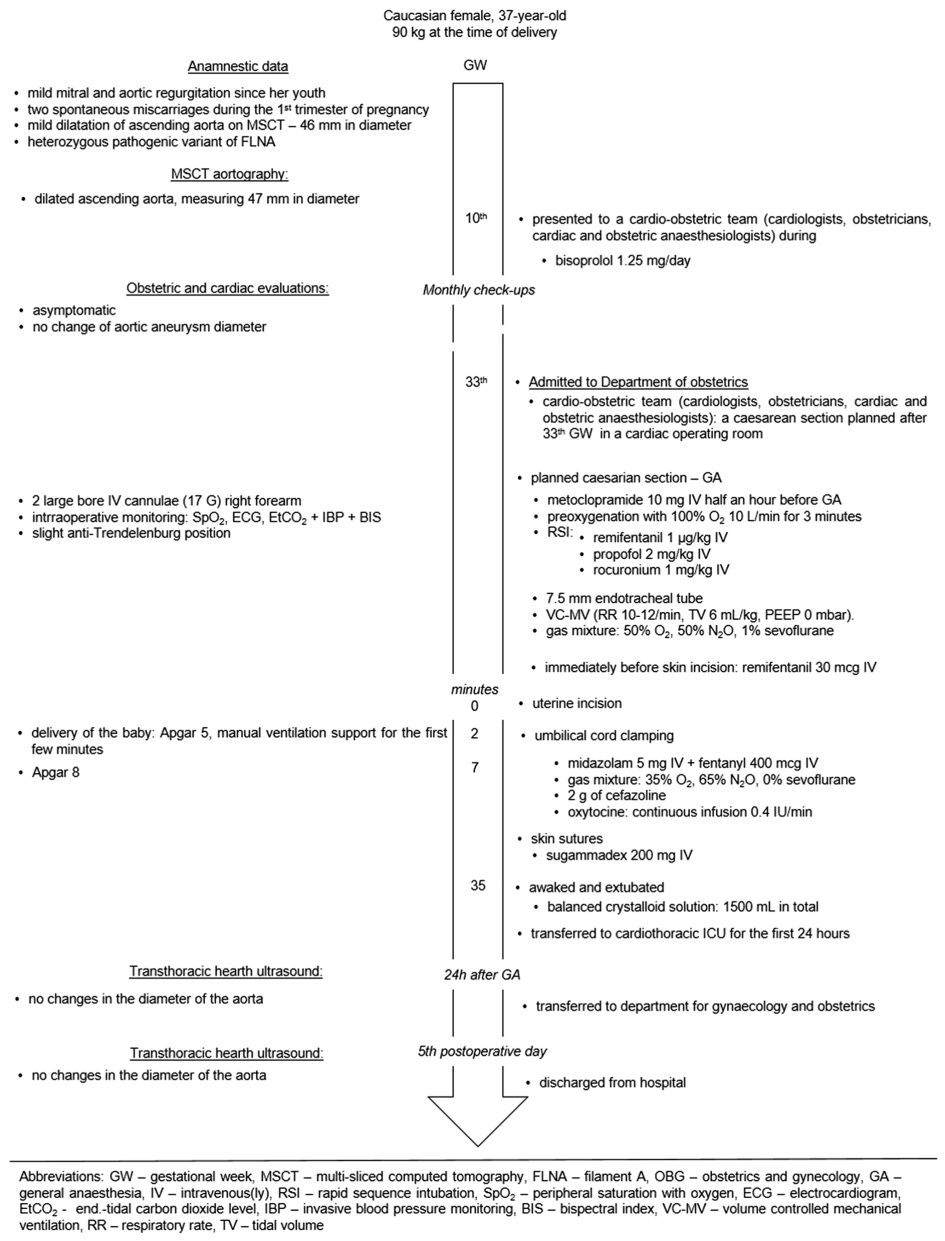
Flowchart of the second parturient with ascending aortic aneurysm

Despite strong advisement against pregnancy due to the significant risks associated, particularly before undergoing repair of the ascending aortic aneurysm, the patient conceived and was presented to the cardio-obstetric team during the 10th GW (Fig. 4). She was prescribed bisoprolol 1.25 mg once daily, and underwent monthly obstetric and cardiac ultrasound examinations. Several months before her pregnancy, a follow-up MSCT aortography revealed a slight increase of the aortic diameter (47 mm, Fig. 2B).

Upon hospitalization in the 33rd week of pregnancy, the cardio-obstetric team promptly decided to proceed with a cesarean section in a cardiac operating room. During the preoperative assessment, we informed the patient that general anesthesia would be administered, due to concerns of potential failure of spinal anesthesia in patients with connective tissue disorders [1–3], as well as the superior ability to control blood pressure during the course of general anesthesia [1–3]. The patient agreed upon the suggested approach. Preparations for the procedure and the administration of general anesthesia mirrored those of the first case (Fig. 4). Two minutes after uterine incision, a premature male infant weighting 2290 g and measuring 43.5 cm in length was delivered. A 1st -minute-Apgar score of 5 necessitated manual ventilation during the first few minutes, with the Apgar score improving to 8 at the 5th minute following birth (Fig. 4).

Following umbilical cord clamping, anesthesia was deepened with midazolam, and adjustments were made to the gas mixture, while sevoflurane discontinued as in the first case (Fig. 4). The patient received antibiotic prophylaxis and a continuous infusion of synthetic oxytocin (Fig. 4.). The intraoperative course proceeded uneventfully, including haemodinamics, Fig. 3B, and BIS values between 40 and 60. At the end of the procedure, neuromuscular blockade was reversed and the patient recovered from anesthesia, and was extubated in the operating room. With stable hemodynamics and adequate respiratory function, she was transferred to the cardiothoracic ICU for further observation during the first 24 h. Since no changes in the diameter of the aorta were detected on transthoracic ultrasound performed on the 1st and 5th postoperative days the patient was allowed for discharge from the hospital.

## Discussion and conclusions

We presented the management of two cases of general anesthesia for cesarean sections in parturients with aortic root dilation exceeding 45 mm. These cases offer numerous clinical points for discussion, some of these will be addressed below.

Despite thoracic aortic aneurysms have an incidence of approximately 6 per 100,000 patient/year [4], the occurrence of ascending aortic aneurysm dissection in pregnant patients is exceedingly rare, accounting for only 0.0004% of all pregnancies [5]. Even though life-threatening complications are rare, around half of all aortic complications in women younger than 40 years of age are associated with pregnancy [5, 6].

Indeed, hemodynamic and hormonal changes during pregnancy contribute to the increased risk of aortic aneurysm [7], with alterations in the cardiovascular system including increased cardiac output (50%), increased heart-rate (10–20 beats/min), and elevated circulating volume and left ventricular mass. Additionally, the aorto-caval compression from the growing fetus raises resistance to aortic outflow, potentially exacerbating the risk of aortic aneurysm dissection and rupture. At the same time, elevated levels of estrogen and progesterone alter the structure of media and intima layers, leading to weakening of the aortic wall [7, 8] These hemodynamic changes peak during the third trimester, coinciding with a significant portion of aortic dissections occurring during pregnancy, with 33% happening in the peri-partal period [8, 9].

In both of our cases, management during pregnancy focused on the reduction of arterial pressure and control of heart rate with the use of a beta-blocker, while anesthesia for cesarean section aimed to mitigate the neuroendocrine response to intubation, thus maintaining hemodynamic stability and minimizing the risks of aortic aneurysm rupture or dissection.

Beta-blockers have been shown to successfully control blood pressure and heart rate and slow the progression of aortic root enlargement [8]. However, not all beta-blockers are safe for use during pregnancy. For instance, atenolol has been associated with fetal malformations and hypospadias when used during the first trimester, whereas metoprolol, propranolol and bisoprolol are deemed safe for use during pregnancy, although regular fetal growth monitoring is recommended [9, 10].

The management of pregnant patients with aortic aneurysm is probably best conducted through a multidisciplinary approach at a tertiary center, involving obstetrician, cardiologist, anesthesiologist, and preferably medical geneticist. Close monitoring, strict blood pressure control and regular heart ultrasound examinations to evaluate of aortic root diameter every 4–8 weeks are crucial [8–10]. Echocardiography is considered to be a safe method, so both of our patients had monthly cardiac evaluations that included cardiac ultrasound examination in addition to regular obstetric checkups.

On the other hand, CT angiography is the gold standard for diagnosing acute aortic dissection [8, 11], with the benefits of an accurate diagnosis outweighing the risks of radiation exposure (0.01–0.66 mGy), which are below the threshold for fetal injury (50 mGy). However, CT angiography was used as a diagnostic procedure before pregnancy in one of the described cases, while in the second case, magnetic resonance angiography was used to diagnose aortic aneurysm during early pregnancy. Currently, there are no studies that have shown any attributable harms of magnetic resonance imaging during any trimester of pregnancy [12].

Surgical repair of known aortic aneurysms and other valvular and aortic conditions meeting certain criteria is recommended prior to pregnancy (which had been also recommended to our patient in the second case), with surgery during pregnancy considered only when conservative treatment fails to control aortic dilation progression (aortic diameter increases too rapidly or exceeds 5 cm, or if aortic valve regurgitation is present) or when the mother’s life is at risk [8–10, 13].

If cardiothoracic surgical repair is indicated before 20 weeks of gestation, termination of pregnancy should be considered because cardiopulmonary bypass increases the risk of long-term neurological impairment and is related to a fetal loss rate of up to 33% [8, 14]. For parturients between 28 and 32 weeks of gestation, cesarean section before surgical repair is indicated because it is believed that risks of exposure of the baby to cardiopulmonary bypass exceeded the risks of premature delivery [8]. In such cases, maternal administration of intravenous magnesium-sulphate for fetal neuro-protection and corticosteroids for lung maturation should be administered [5]. For pregnancies at approximately 25 GW, surgical repair with the fetus remaining in utero, is thought to be the treatment of choice [11, 14].

Considerable hemodynamic changes are present during labor and the early post-partal period, since approximately 500 mL of venous blood is forced backwards with each uterine contraction. Pain and Valsalva maneuvers during pushing efforts significantly contribute to central venous pressure fluctuations. At the third stage of labor, the delivery of the placenta may lead to a situation of “autotransfusion” of an additional 500 mL of blood from the utero-placental into the maternal circulation. These changes also result in an increased central venous pressure, preload, and cardiac output, with a concomitant increase of the risk of aortic aneurysm rupture [15].

The mode of delivery in parturients with aortic aneurysm depends on factors such as aneurysm size, presence, or absence of aortic dissection, a possible underlying aortopathy and fetal gestational age [8]. For low-risk patients having an aortic diameter less than 40 mm, vaginal delivery is recommended, however, strict blood pressure and heart-rate control are still of great importance to decrease the risk of dissection or aortic rupture. Blood pressure and heart-rate are usually maintained by adequate analgesia via an epidural catheter [7, 16].

Cesarean section is typically recommended for parturients with an aortic diameter exceeding 45 mm, with careful attention to maintaining hemodynamic stability throughout the procedure, regardless of whether general or regional anesthesia is employed [7, 9, 16]. For both of our cases obstetricians opted for caesarian delivery, which were both conducted in a cardiac operating room. Since cardiac operating rooms are not well equipped for caesarian sections, all necessary obstetric surgical instruments and necessities were prepared for the caesarian section, while a neonatology table was preheated and ready for the neonate. The caesarian sections were performed by an obstetric team and a neonatology team with neonatal resuscitation equipment ready for the newborn, and the neonatal intensive care unit being informed about a possible admission. In addition, the cardiac surgery team and perfusionist (including their equipment) were ready in case of necessity.

Although regional anesthesia is a method of choice for cesarean section, general anesthesia (GA) is usually conducted in high-risk parturients, such as those suffering from preeclampsia or heart and vascular diseases [17–19]. Traditionally, for induction of GA in pregnant women, a rapid-sequence induction (RSI) with a hypnotic agent and a fast-acting muscle relaxant is used. However, the usual absence of opioid use during induction, to avoid respiratory depression in a neonate since they readily cross the placental barrier, leads to a strong neuroendocrine response that includes a severe increase in blood pressure and heart-rate [18, 20]. However, the main objective of anesthetic management during cesarean section in parturients with ascending aortic aneurysm is to maintain hemodynamic stability throughout the procedure [18, 21, 22], while attenuating the hemodynamic response to endo tracheal intubation and surgical stimulation without inducing respiratory depression in the neonate.

Several randomized placebo-controlled studies have investigated the effects of remifentanil on maternal hemodynamic variables and neonatal outcome with conflicting results, in our opinion mainly due to the differences in remifentanil dosage [17, 19, 21, 23–25]. However, the meta-analysis done in 2019 reported that the use of remifentanil probably maintains hemodynamic stability during the procedure, despite the conflicting results mentioned previously [22]. In our cases remifentanil was found to be a proper choice for maintaining hemodynamic stability.

Usually, the elimination of remifentanil from the newborn’s circulation at the time of birth is considered to be nearly completed due to its short duration of action [26]. However, all three of the newborns in our cases needed manual ventilation support during first minutes of life. Improvement in Apgar score was seen within 5-min after delivery and then, no further need for manual ventilation was necessary. Considering that the elimination half-life of remifentanil is 3–10 min, it is possible that remifentanil was not eliminated from the neonatal circulation at the time of delivery.

Although there are no studies investigating the conduction of general anesthesia for cesarean sections for parturients with aortic aneurysms, three published case reports described the successful use of remifentanil during general anesthesia for cesarean section in parturients with aortic aneurysm in Marfan syndrome [27–29]. In these three case reports, the authors reported the hemodynamic stability of parturients during the whole procedure, similar to our patients. On the other hand, Singh reported the need for neonatal ventilation one minute after delivery [27], as we reported, while the babies in Miyawaki’s [28] and Young’s reports [29] did not need for respiratory support.

Another way to avoid the respiratory depression in neonate is to reduce the use of opioids in parturient, which could be done by applying neuroaxial, instead of general anesthesia. Neuroaxial modes (spinal and epidural anesthesia) are reported to be preferred types of anesthesia for caesarean section, including for patients with aortic lesions [30], as in our patients. The first choice for both patients would thus be providing a spinal anesthesia. However, the first patient refused it and in the second we decided to conduct general anesthesia, mainly because of concerns on failure of spinal anesthesia in patients with connective tissue disorders [1–3]. Regardless of the type of anesthesia, it is priority to maintain hemodynamic stability by avoiding an increase of systemic vascular resistance (which increase aortic sheer stress) and hypotension (which endangers fetal perfusion) [30].

Except of the hypotension induced by anesthesia and hypertension due to the pain provoked by intubation and surgical stimulus, the application of uterotonics during the caesarean section is another important factor needing consideration when managing the parturient with ascending aortic aneurysm. Indeed, uterotonics are known to induce hypertension, with ergometrine having additional pronounced hypertensive and spastic effect on coronary arteries as well, which could even lead to myocardial infarction [31, 32]. Therefore, guidelines recommend avoiding ergometrine, with use oxytocin instead [10].

In our patients there were no hypertensive period during the operation and they did not need intervention of cardiac surgery team during and immediately after the caesarean section was performed. In addition, to ensure that there is no increase in aortic aneurism diameter we could use transoesophageal echocardiography (TOE) during the surgery. TOE during caesarean section in parturients with Marfan’s syndrome has been reported [27, 33]. However, we did not opt for it. Despite TOE is being considered safe and is the preferred imaging method in pregnancy [10], there is a risk of vomiting and aspiration, as well as a sudden increases in intra-abdominal pressure [10], which are not welcomed during the anesthesia nor abdominal surgery, especially when it could compromise the fetal circulation. Therefore, in both cases we relied solely on clinical status and monitoring (Figs. 1 and 4) with the addition of invasive blood pressure monitoring.

Overall, the management of parturients with ascending aortic aneurysm is complex and necessitates a comprehensive approach involving various medical specialties, including cardiologist, obstetrician, cardiac surgeon and anesthesiologist. If an ascending aortic aneurysm is diagnosed during the pregnancy or it was not repaired before the pregnancy as it is recommended, elective cesarean delivery in tertiary centers with available cardiac surgery and blood products is obligatory, to be prepared in cases of catastrophic events, such as aortic dissection or rupture during the perioperative period.

In the context of preoperative assessment, communication with patients regarding the risks and benefits of different anesthesia options is crucial. General anesthesia with administration of remifentanil is a reasonable choice in these cases because it enables the blunting of the hypertensive response to intubation and surgical stimulation. Due to placental transfer of remifentanil, neonatal respiratory depression is to be expected during the first minutes after delivery, especially if larger bolus dose of remifentanil is used. We consider maintenance of haemodynamic stability to be paramount in the prevention of a rupture or dissection of an ascending aortic aneurysm during delivery of a newborn.

## Data Availability

Data sharing is not applicable to this article as no datasets were generated or analyzed during the current study.

## Abbreviations

GA: General anesthesia
RSI: Rapid-sequence induction
GW: Gestational week
US: Ultrasound
IV: Intravenous(ly)
BIS: Bispectral (index)
ICU: Intensive care unit
CT: Computed tomography
FLNA: Filamin A
